# Patient-Specific Identification of Atrial Flutter Vulnerability–A Computational Approach to Reveal Latent Reentry Pathways

**DOI:** 10.3389/fphys.2018.01910

**Published:** 2019-01-14

**Authors:** Axel Loewe, Emanuel Poremba, Tobias Oesterlein, Armin Luik, Claus Schmitt, Gunnar Seemann, Olaf Dössel

**Affiliations:** ^1^Institute of Biomedical Engineering, Karlsruhe Institute of Technology (KIT), Karlsruhe, Germany; ^2^Medizinische Klinik IV, Städtisches Klinikum Karlsruhe, Karlsruhe, Germany; ^3^Institute for Experimental Cardiovascular Medicine, University Heart Center Freiburg Bad Krozingen, Freiburg, Germany; ^4^Faculty of Medicine, Albert-Ludwigs University, Freiburg, Germany

**Keywords:** atrial flutter, ablation, vulnerability, computational modeling, personalized model

## Abstract

Atypical atrial flutter (AFlut) is a reentrant arrhythmia which patients frequently develop after ablation for atrial fibrillation (AF). Indeed, substrate modifications during AF ablation can increase the likelihood to develop AFlut and it is clinically not feasible to reliably and sensitively test if a patient is vulnerable to AFlut. Here, we present a novel method based on personalized computational models to identify pathways along which AFlut can be sustained in an individual patient. We build a personalized model of atrial excitation propagation considering the anatomy as well as the spatial distribution of anisotropic conduction velocity and repolarization characteristics based on a combination of a priori knowledge on the population level and information derived from measurements performed in the individual patient. The fast marching scheme is employed to compute activation times for stimuli from all parts of the atria. Potential flutter pathways are then identified by tracing loops from wave front collision sites and constricting them using a geometric snake approach under consideration of the heterogeneous wavelength condition. In this way, all pathways along which AFlut can be sustained are identified. Flutter pathways can be instantiated by using an eikonal-diffusion phase extrapolation approach and a dynamic multifront fast marching simulation. In these dynamic simulations, the initial pattern eventually turns into the one driven by the dominant pathway, which is the only pathway that can be observed clinically. We assessed the sensitivity of the flutter pathway maps with respect to conduction velocity and its anisotropy. Moreover, we demonstrate the application of tailored models considering disease-specific repolarization properties (healthy, AF-remodeled, potassium channel mutations) as well as applicabiltiy on a clinical dataset. Finally, we tested how AFlut vulnerability of these substrates is modulated by exemplary antiarrhythmic drugs (amiodarone, dronedarone). Our novel method allows to assess the vulnerability of an individual patient to develop AFlut based on the personal anatomical, electrophysiological, and pharmacological characteristics. In contrast to clinical electrophysiological studies, our computational approach provides the means to identify all possible AFlut pathways and not just the currently dominant one. This allows to consider all relevant AFlut pathways when tailoring clinical ablation therapy in order to reduce the development and recurrence of AFlut.

## 1. Introduction

The long-term success rate of atrial fibrillation (AF) ablation is unsatisfactory low, particularly in patients suffering from persistent AF. Besides AF recurrence, the development of post-ablational atrial flutter (AFlut) represents a major problem (Villacastín et al., 2003; Kobza et al., 2004; Chugh et al., 2005; Patel et al., 2008; Yamada and Kay, 2013; Biviano et al., 2015). In more than half of the patients, sustained AF is reinitiated within 5 years after ablation or AFlut develops (Bunch et al., 2016). 20% of recurrences after AF ablation in elderly are due to AFlut (Dong et al., 2015). In the general AF population, 18.5% of patients were diagnosed with AFlut during a median follow-up time of 421 day post ablation (Gucuk Ipek et al., 2016). Liang et al. (2015) observed AF or organized atrial tachycardia in 53% of 300 patients within the first 6 weeks after pulmonary vein (PV) isolation. AF and AFlut are often even combined endpoints in studies evaluating the success of AF ablation (Bunch et al., 2016). Waldo and Feld (2008) highlighted the inter-relationship between AF and AFlut. AF precedes AFlut in most cases forming the required line of block by fibrillatory conduction. Moreover, ablation of atrial tissue can lead to a substrate for AFlut. Particularly gaps in linear lesions forming isthmuses or revitalized tissue areas forming zones of slow conduction render the atria vulnerable. Also PV isolation has been associated with a substantial risk to develop AFlut (Mesas et al., 2004; Deisenhofer et al., 2006; Jaïs et al., 2006). Castrejón-Castrejón et al. (2011) reviewed the occurrence of organized atrial tachycardia such as AFlut after AF ablation and emphasize that more extensive left atrium (LA) ablation renders the patient more vulnerable to AFlut. However, the exact origin of the pathologic substrate is not understood. Therefore, we present a method to assess the vulnerability to AFlut in personalized computational models. Besides an identification of possibly AFlut sustaining pathways in the observed state of the patient (*baseline*), the approach allows to assess the effect of different therapeutic strategies such as ablation patterns, pharmacological compounds, or other anatomical and electrophysiological interventions *in silico* before actually performing them *in vivo*.

A common approach to simulate complex excitation patterns is the monodomain reaction-diffusion model which is based on the transport of ions within the domains and across the cell membrane (Niederer et al., 2011). As such, it considers electrotonic effects and source-sink relations resulting in e.g., convex or concave wavefronts. On the one hand, the monodomain approach provides the means to study complex and chaotic patterns such as fibrillation including wave breaks. On the other hand, the monodomain model is computationally expensive even using optimized implementations (Labarthe et al., 2014; Pezzuto et al., 2017). Hence, a thorough exploration of the parameter space regarding effects on the three-dimensional whole organ level, as e.g., the vulnerability to arrhythmia caused by ectopic stimuli from a multitude of locations and at varying time instants, is infeasible. Eikonal approximations of the continuous dynamics of the reaction-diffusion system allow to simulate excitation propagation in terms of activation times with significantly reduced computational load by several orders of magnitude (Wallman et al., 2012; Labarthe et al., 2014; Loewe, 2016; Neic et al., 2017; Pezzuto et al., 2017) as only one static, non-linear partial differential equation derived from the monodomain model has to be solved, which makes it interesting for simulations of cardiac activation (Keener, 1991; Franzone and Guerri, 1993). In contrast to level set methods in general, shortest pathway (van Dam and van Oosterom, 2003) and fast marching methods assume monotonously expanding wavefronts. Thus, a specific approach considering multiple fronts, reentry, and anisotropic conduction was developed for cardiac electrophysiology (Sermesant et al., 2007; Pernod et al., 2011) based on the fast marching method on structured grids (Sethian, 1996, 1999; Sermesant et al., 2005). Several extensions provide the means to consider wavefront curvature and the mesh structure if that is needed for the specific application (Sethian and Vladimirsky, 2000, 2003). Ablation of ventricular tissue in order to prevent scar-related ventricular tachycardia was presented as a potential application for this method (Pernod et al., 2011). However, one should keep in mind that while current multifront fast marching methods faithfully represent macro-reentrant arrhythmias like AFlut, they are not well suited to study more complex reentries like spiral wave or multiple wavelet reentry where local source-sink mismatch plays a crucial role (Loewe, 2016).

The aim of this work is to develop a method that allows to comprehensively assess the vulnerability to AFlut in personalized models considering both anatomical and electrophysiological properties allowing to evaluate therapeutic approaches such as ablation and drug treatment *in silico*.

## 2. Materials And Methods

**Figure 1:**
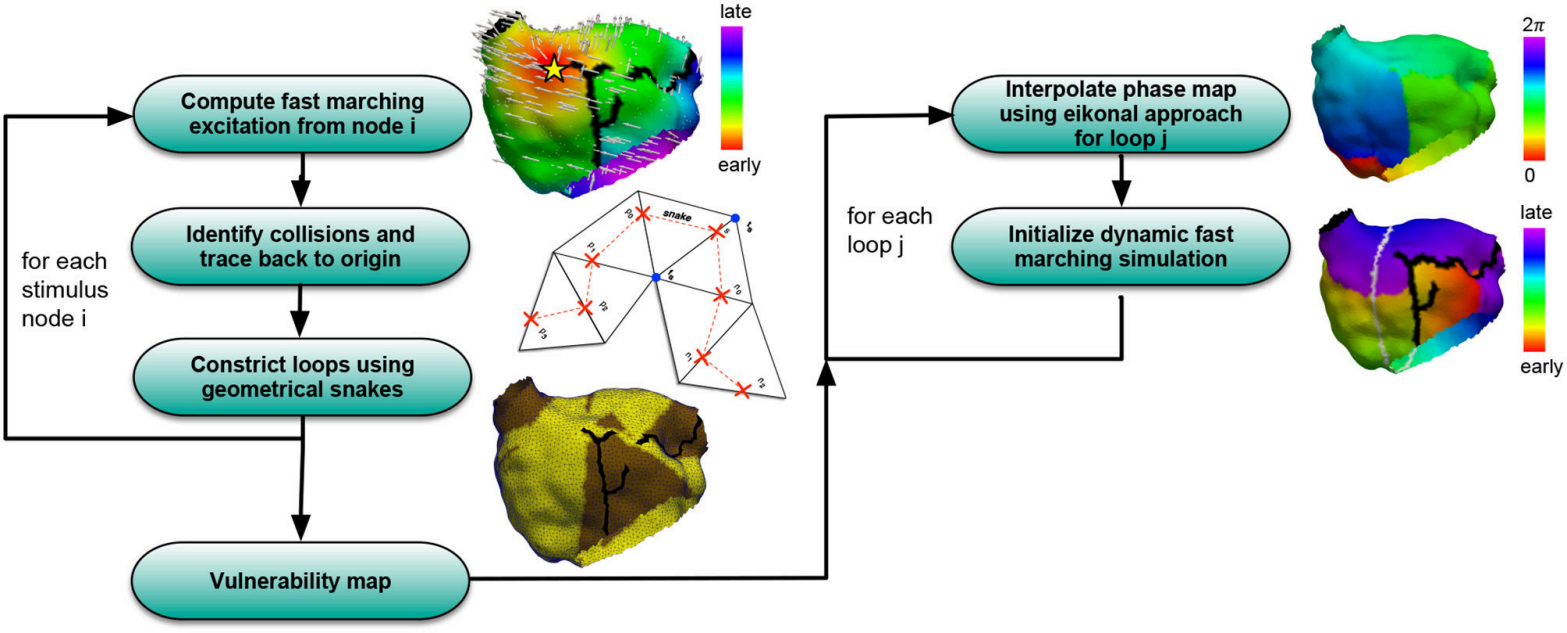
Overview of the algorithm to compute vulnerability maps considering all possible AFlut loops (left) and transfer the AFlut scenarios to dynamic simulations (right).

A simulation pipeline consisting of several steps (Figure 1) was developed in order to assess the vulnerability to AFlut. In this section, the different building blocks of the workflow are presented.

### 2.1. Fast Marching Simulation of Excitation Propagation

The eikonal equation governs the spread of an activation wave in a possibly anisotropic medium resulting in a scalar field *t*_*a*_(*x*_*i*_) – the activation map:

(1) $$c\sqrt{\nabla t_{a}\vec{G}\nabla t_{a}}=1,$$

with *c*(*x*_*i*_) being the speed function defined for each node *x*_*i*_, *t*_*a*_(*x*_*i*_) being the activation time, and $\vec{G}$ being a tensor enabling anisotropy to account for faster conduction along the principal axis of myocytes than perpendicular to it (Loewe, 2016):

(2) $$\begin{array}{r} \vec{G}=R\left(\phi ,\theta \right)\left(\begin{array}{ccc} k & 0 & 0\\ 0 & 1 & 0\\ 0 & 0 & 1 \end{array}\right)R{\left(\phi ,\theta \right)}^{T}\;, \end{array}$$

with *R(ϕ, θ)* rotating the coordinate system to align the positive x-axis with the prinicipal myocyte orientation. The multifront fast marching scheme for the eikonal-based simulation of excitation propagation introduced by Sermesant et al. (2007) (Supplementary Algorithm 1) was extended to consider restitution of conduction velocity (CV) and effective refractory period (ERP). Restitution of both parameters with respect to basic cycle length (BCL) was determined by pacing in a one-dimensional tissue strand using the Courtemanche et al. cell model (Courtemanche et al., 1998) coupled in a monodomain approach as detailed in Wilhelms et al. (2013). These restitution curves provide opportunities for functional model personalization in the future as further discussed below. The resulting curves for CV were approximated by fitting to exponential decays:

(3) $$CV\left(BCL\right)=A-B\cdot \exp\left(-\frac{BCL}{C}\right)\;.$$

The BCL was defined as the time passed since the last activation of the respective node and initialized with a user-defined value either globally or for each node individually. The restitution of the ERP was described the same way.

### 2.2. Identification of Flutter Loop Candidates

The fast marching approach was used to trigger excitations from a multitude of locations sequentially. For each stimulus location, activation times were computed and stored together with information regarding the spread of excitation in terms of a vector pointing from the activating to the activated node. Wavefront collision sites were defined as points of latest activation on circular pathways composed of two traces originating from the stimulus site to opposite sides, i.e., at an angle of approximately π. On the one hand, these pathways are the shortest in the sense of wave propagation, i.e., they are not artificially prolonged by zig-zag patterns but determined as the shortest connection by the fast marching algorithm. On the other hand, they are locally the longest as two independent waves collided on the loop. A wavefront collision for node *i* was identified if the following condition was fulfilled for any neighboring node *j*:

(4) $${\frac{\vec{a_{i}}}{∥\vec{a_{i}}∥}}_{2}+{\frac{\vec{a_{j}}}{∥\vec{a_{j}}∥}}_{2}<0.99\;,$$

with $\vec{a_{i}}$/$\vec{a_{j}}$ being the vector pointing from the node that activated node *i*/*j* to node *i*/*j* itself. The condition identifies all points at which the vectors meet at an angle ∈ (π/2,3π/2), thus pointing in opposite directions. From the sites of collisions, loops were defined by the two traces along the steepest negative activation time gradient leading back to the stimulus location. A loop was thus composed of a circular, ordered series of nodes. Along the loop, the round trip time (RTT) was calculated considering the heterogeneous and anisotropic tissue properties in terms of CV. If a loop did not fulfill the wavelength (WL) condition

(5) $$\begin{array}{r} \underset{i}{\max}\left(ERP_{i}\left(RTT\right)\right)<RTT, \end{array}$$

it was disregarded. Here, *ERP*_*i*_ is the ERP of node *i* considering a BCL equal to the RTT according to Equation (3). *i* iterates over all nodes spanning the loop candidate.

### 2.3. Constriction of Flutter Loop Candidates

The fact that the loops were traced back all the way to the initial stimulus site introduced artifacts as a dynamic wave would cut short between the two traces from the site of collision to the stimulus site in many cases. In the easier case, both half loops share a part of the loop. Under such circumstances, all common nodes can be neglected, thus shrinking the loop (Supplementary Figure 1A). In most cases, however, artifacts of other types were present as well. In Supplementary Figure 1B, a shortcut of the two half loops running adjacent on the posterior LA wall can be anticipated between the posterior interatrial connections and the connection via the coronary sinus. Therefore, a geometric snake approach (Supplementary Figures 2, 3) considering anisotropy was implemented in order to constrict the loops like a rubber band by minimizing the spline energy. Evolving snakes on triangular meshes were proposed before for mesh scissoring operations and constriction detection (Lee and Lee, 2002; Hétroy and Attali, 2003; Bischoff and Kobbelt, 2004; Lee et al., 2004) and were adapted to the requirements of the specific application in this work as further detailed in the Supplementary Methods.

### 2.4. Eikonal-Diffusion Phase Extrapolation

**Figure 2:**
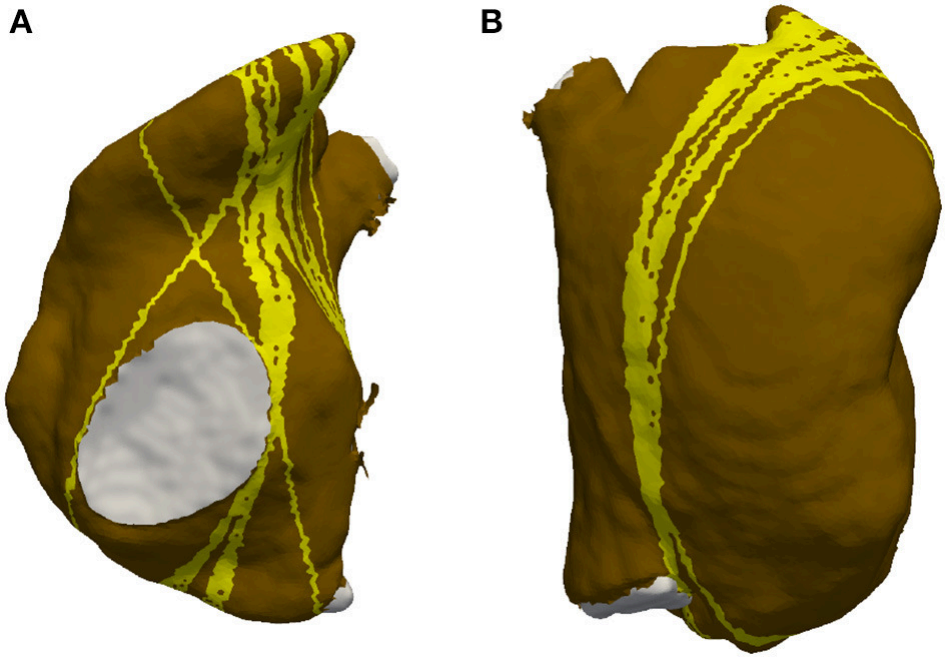
Example of an AFlut vulnerability map for the right atrium (anterior aspect in **A**, posterior aspect in **B**). The vulnerable pathways are marked in yellow on the substrate of the right atrium (brown); the blood pool is indicated in gray. Several pathways run from the septal side of the tricuspid valve to the right atrial appendage.

The methods introduced above allow to identify pathways in an atrial model that can potentially sustain AFlut. However, the pathways are not necessarily dominant and might thus not be expressed in dynamic scenarios. An example is shown in Figure 2 where several pathways run from the septal side of the tricuspid valve to the right atrial appendage and to the coronary sinus region. Each pathway is locally the shortest and long enough to sustain AFlut according to the WL condition. However, according to Huygen's principle, only one pathway will dominate the excitation pattern distal to the constriction at the tricuspid valve where all pathways narrow. Thus, the remaining pathways will be suppressed. In order to identify the dominant pathway, i.e., to distinguish between theoretically vulnerable pathways and practically inducible pathways, the initial state for a dynamic simulation can be extrapolated from a single loop to the entire simulation domain as detailed in the Supplementary Methods.

Figure 1 summarizes the pipeline used to generate AFlut vulnerability maps and transfer the results to dynamic simulations.

### 2.5. Heterogeneous Tissue Properties, Disease-Specific Substrates, and Drug Effects

**Figure 3:**
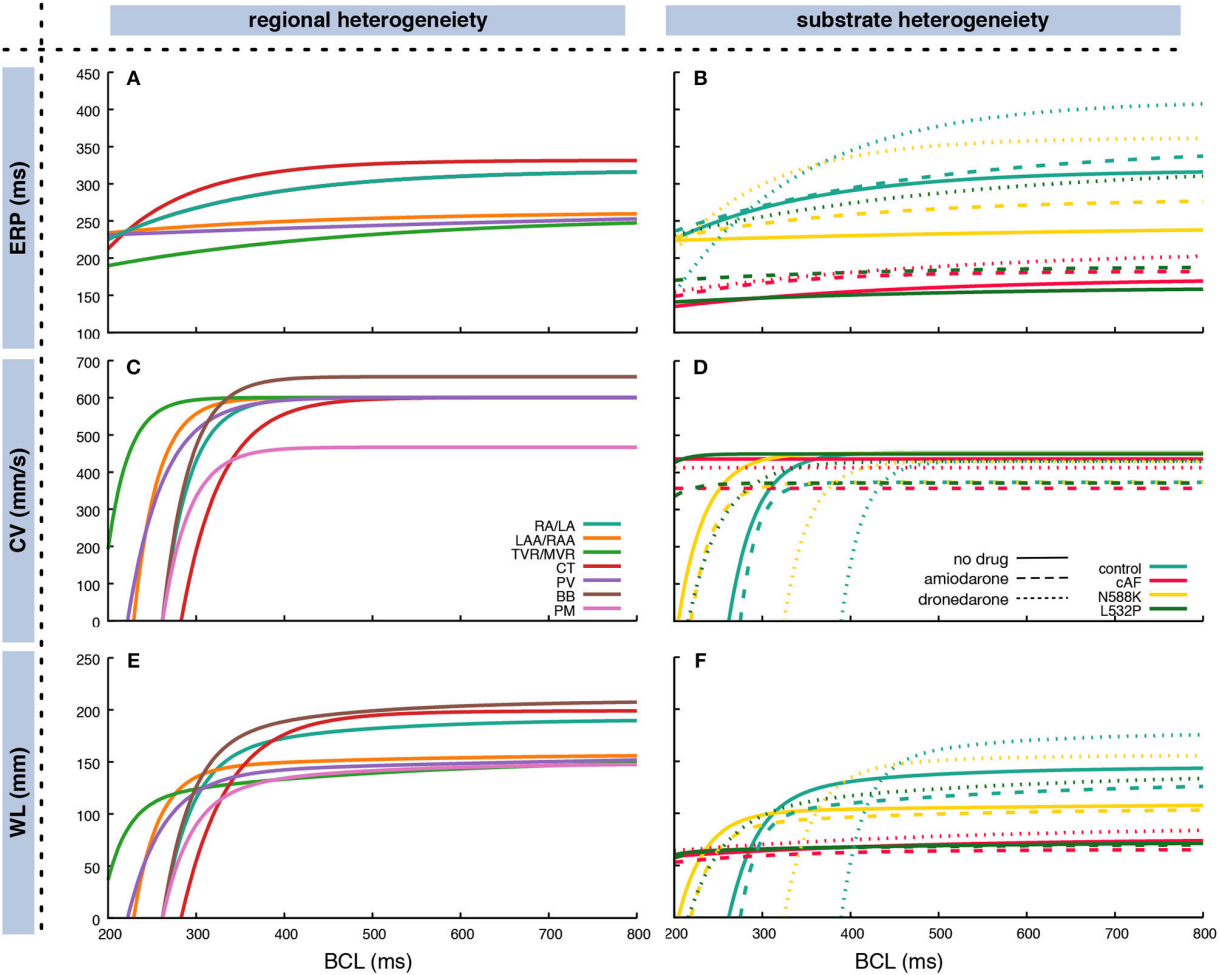
Fitted exponential restitution curves of ERP **(A,B)** and CV **(C,D)**, as well as the WL as the product of the former measures **(E,F)** for different anatomical regions in the atria **(A,C,E)** and different substrates **(B,D,F)**. In **(B,D,F)**, the dashed lines represent the respective substrates under the influence of 2.3 μM amiodarone whereas the dotted lines represent the influence of 0.21 μM dronedarone. Exponential curves according to Equation (3) were used to fit the output of monodomain tissue strand simulations. The CV for the homgeneous control model in **(D)** was reduced compared to the RA/LA tissue in the heterogeneous setup in **(C)** to obtain a similar total atrial activation time. In **(A)**, BB and PM curves are covered by the RA/LA curve. Coefficients are listed in Supplementary Table 1. RA, right atrium; LA, left atrium; CT, crista terminalis; PM, pectinate muscles; BB, Bachmann's bundle; II, inferior isthmus; PV, pulmonary veins; RAA, right atrial appendage; LAA, left atrial appendage; TVR, tricuspid valve ring; MVR, mitral valve ring.

The restitution of CV and ERP was determined through monodomain simulations in a one-dimensional tissue strand as detailed in Wilhelms et al. (2013). CV and ERP were determined for 50 BCLs between 200 and 1,000 ms distributed linearly in frequency domain. Regional heterogeneity between different anatomical areas within the atria was accounted for as described previously in terms of both electrophysiological properties (Krueger et al., 2013) and monodomain conductivities (Loewe et al., 2015) in a heterogeneous setup. Furthermore, four different atrial substrates were analyzed in homogeneous setups: (i) a control substrate representing healthy myocardium modeled by the original Courtemanche et al. (1998) cellular model, (ii) a substrate which has undergone remodeling due to chronic atrial fibrillation (cAF) (Loewe et al., 2014b), (iii) a substrate with the N588K mutation in the human ether-à-go-go-related gene (hERG) (Loewe et al., 2014c), and (iv) a substrate with hERG mutation L532P (Loewe et al., 2014c). The latter two substrates have been associated with familial AF and are used here to demonstrate how patient-specific information like genotype-specific repolarization properties can be included in the overall workflow. All four substrates were investigated with and without the influence of two exemplary antiarrhythmic drugs to demonstrate how not only ablation but also drug therapy can be considered and evaluated using our method. Based on a previous study (Loewe et al., 2014a), the class III antiarrhythmic compounds amiodarone and dronedarone where chosen and modeled as detailed there. Figure 3 shows the exponential fit of the restitution curves based on the coefficients in Supplementary Table 1. The CV for the homgeneous control model was reduced compared to the RA/LA tissue in the heterogeneous setup to obtain a similar total activation time.

The ERP for long BCLs ranged between 256 ms for the tricuspid and mitral valve rings to 332 ms for the crista terminalis. Crista terminalis, Bachmann's bundle, and the working myocardium showed a steeper decrease toward shorter BCLs compared to the remaining regions (Figure 3C). Regarding AFlut vulnerability, the WL is the decisive factor. Both, different regions and different substrates, exhibited distinct behavior at different BCLs. For example, at short BCLs, crista terminalis was the region with the shortest WL opposed to long BCLs where it was the region with the longest WL together with Bachmann's bundle (Figure 3E).

### 2.6. Clinical Example

The proposed method was applied to a clinical example from a 70 year-old female patient who underwent electroanatomical mapping due to atypical AFlut after previous ablation for AF. The previous AF ablation comprised PV isolation, ablation of a mitral isthmus line and the cavotricuspid isthmus. The patient presented with atypical AFlut with a cycle length of 420 ms. Mapping was performed using the Rhythmia system (Boston Scientific, Marlborough, MA, USA). The PVs were still isolated, electrogram voltage along the mitral isthmus line was reduced but the line was not blocked. A zone of slow conduction was identified on the left anterior wall close to the left PVs. Five different AFlut types with cycle lengths between 280 and 470 ms could be induced clinically. The arrhythmia terminated to sinus rhythm upon the first ablation in the zone of slow conduction. Further ablation points were placed in the area of the anterior line and connected to the mitral valve. Afterwards, tachycardia could not be induced anymore by burst pacing from the coronary sinus with cycle lengths down to 200 ms. The LA geometry aquired during the procedure comprising 7,471 nodes was retrospectively exported from the clinical system and transferred to the fast marching simulation environment. The previous ablations were manually annotated in the patient LA geometry (Figure **10A**). To reproduce the clinical reentry pattern and activation map qualitatively, the CV was homogeneously set to 650 mm/s and the ERP to 250 ms. The protocol was approved by the ethics committee of the University of Freiburg. The subject gave written informed consent in accordance with the Declaration of Helsinki.

## 3. Results

### 3.1. Flutter Loops and Geometric Snakes

**Figure 4:**
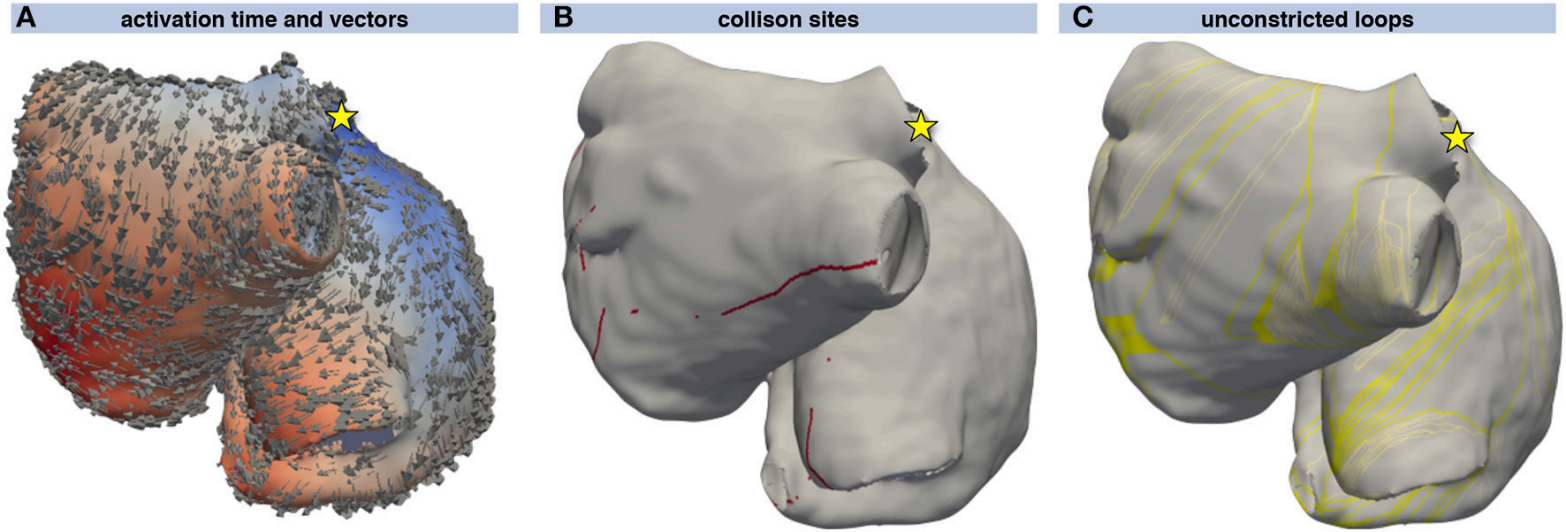
Loop candidate finding. Activation time resulting from a stimulus at the junction of the superior vena cava with the right atrial appendage (yellow star) ranging from early (blue) to late (red). The direction of activation is indicated by arrows **(A)**. Points of wavefront collisions were detected and are indicated by red dots in **(B)**. **(C)** shows the loops composed of the two traces leading from the collision site to the stimulus site (yellow star). The yellow loops fulfill the WL condition Equation (5) whereas the light gray loops do not and were thus not considered for further steps.

Excitation propagation was calculated from several stimulus locations and sites of collision were detected using the activation vectors as shown in Figures 4A,B. From the sites of wavefront collision, the activation front was traced back to the stimulus site along the gradient of the activation time field. Combining the traces obtained by following the activation waves of both colliding waves yielded a set of initial loops for each stimulus node (Figure 4C). The WL condition Equation (5) was not fulfilled by several loops that could thus be neglected during the following steps (lighter colored loops in Figure 4C).

**Figure 5:**
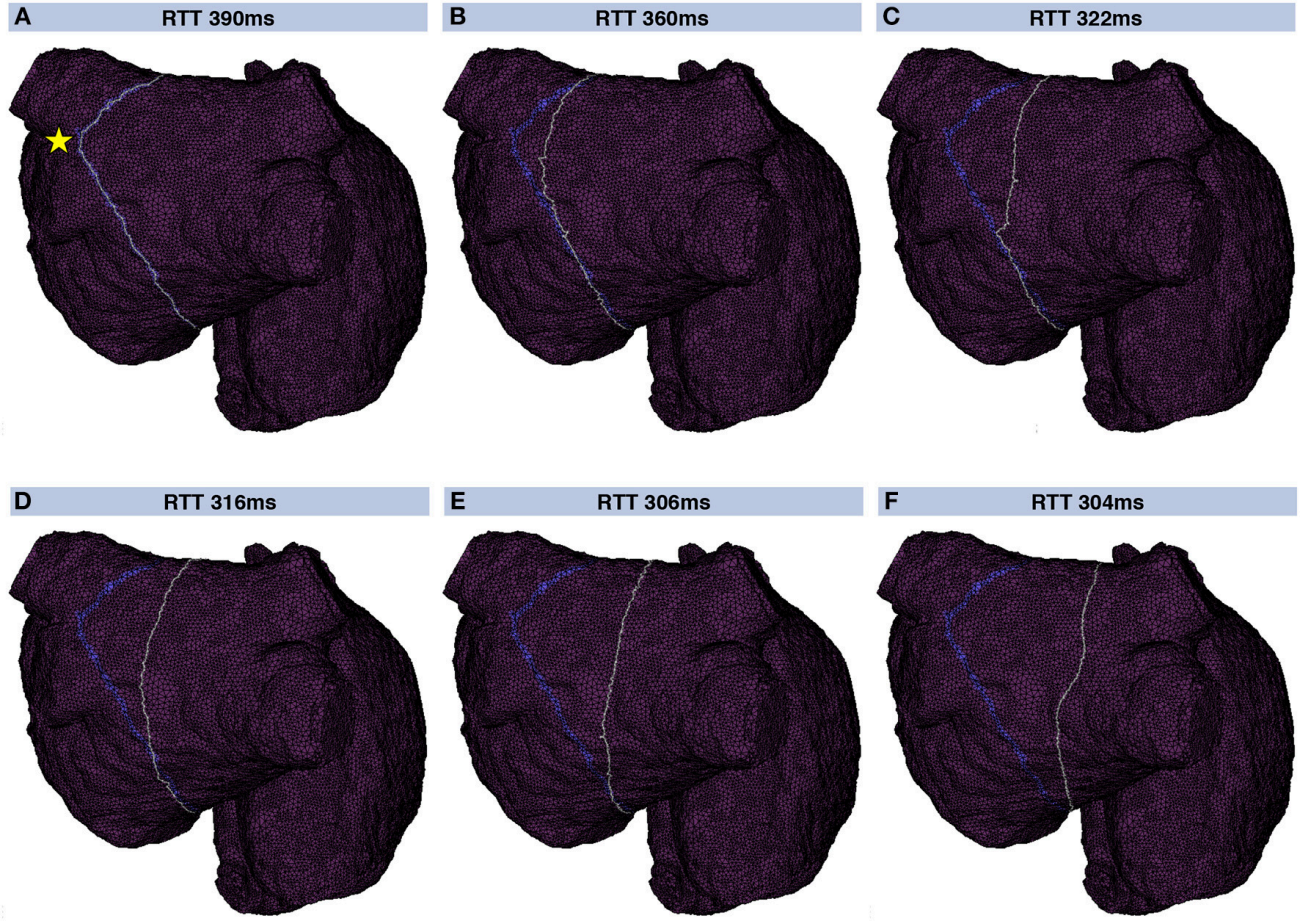
Evolution of a geometric snake covering the LA. Initially, the snake (gray) covered the loop found by the collision tracing algorithm (blue band) corresponding to a RTT of 390 ms **(A)**. By iteratively constricting the snake **(B–F)**, the shortest RTT of 304 ms considering heterogeneous CV and anisotropy was found. In this way, the influence of the particular choice of the stimulus site (yellow star in **A**) was reduced.

A geometrical snake was initialized for each valid loop candidate. Figure 5 shows a geometrical snake initialized along a flutter loop candidate in the LA. The stimulus leading to that loop was applied between the two left PVs (yellow star in Figure 5A). The segment connecting the loop candidate with the stimulus location was shared by both half loops and disregarded before the snake was initialized. The initial RTT of 390 ms was reduced to 304 ms by iterating the snake according to Supplementary Equation (4). The converged snake reflects myocyte orientation and CV heterogeneity (Figure 5F).

### 3.2. Vulnerability Maps

**Figure 6:**
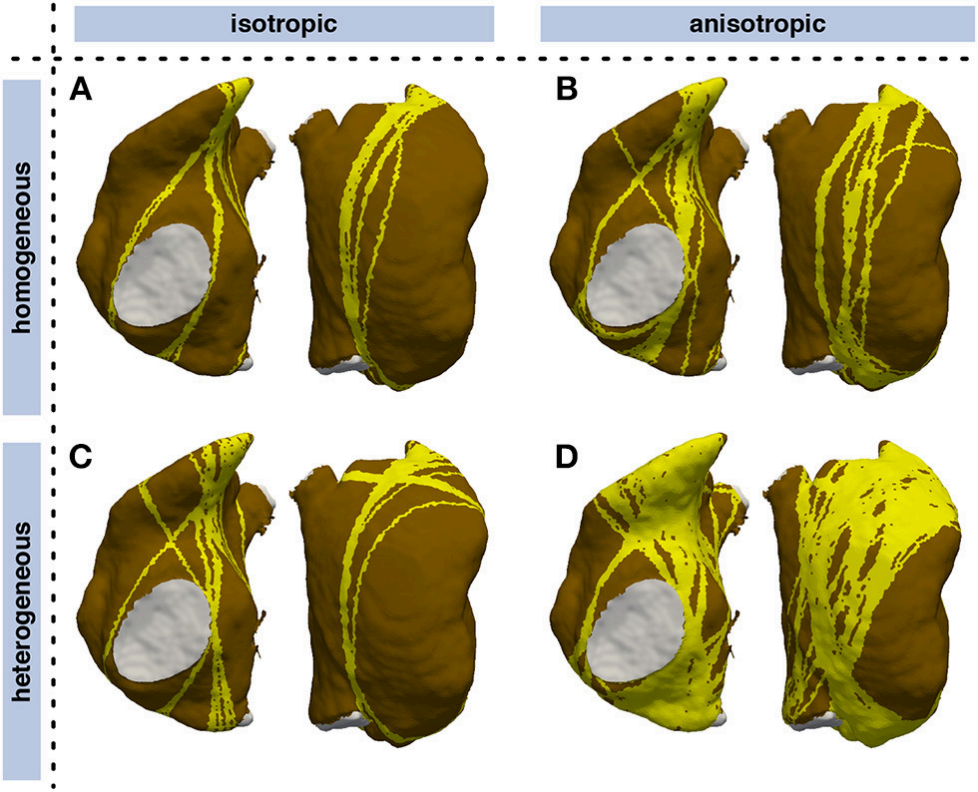
Influence of heterogeneity and anisotropic conduction on vulnerability maps of the RA. Vulnerable pathways are marked in yellow on the RA myocardium (brown); the blood pool is indicated in gray. While CV was tuned such that the latest sinus rhythm activation coincided in all models, both anisotropy and heterogeneity lead to a higher number of vulnerable pathways. **(A)** isotropic homogeneous; **(B)** anisotropic homogeneous; **(C)** isotropic heterogeneous; **(D)** anisotropic heterogeneous.

By triggering stimulation from different points, identifying loop candidates, and constricting them using geometrical snakes, AFlut vulnerability maps were generated as outlined in Figure 1. In a first step, an MRI-derived triangular mesh of the RA comprising 19,296 nodes augmented with rule-based tissue labels and myocyte orientation (Wachter et al., 2015) was used. The triangular surface model is a lumped representation of the atrial wall with a single myocyte orientation in each element. As detailed in Wachter et al. (2015) and Loewe (2016), crista terminals and the pectinate muscles were integrated within the RA wall. The vulnerability maps were sensitive to tissue anisotropy as indicated by the lower number of flutter pathways in the isotropic models (Figures 6A,C) compared to the anisotropic cases (Figures 6B,D). While the heterogeneous *A* and *B* values defining the CV according to Equation (3) were scaled in the isotropic and homogeneous cases to match the sinus rhythm activation time of the last element in the heterogeneous anisotropic simulation, both anisotropy and heterogeneity increased the number of vulnerable pathways. In the homogeneous isotropic setup (Figure 6A), only 12.7% of all elements were covered by vulnerable pathways. Adding anisotropy (Figure 6B) increased the coverage to 20.8% whereas adding heterogeneity (Figure 6C) lead to 15.9% coverage. 51.7% of the fully heterogenous and anisotropic model (Figure 6D) were covered by vulnerable pathways.

**Figure 7:**
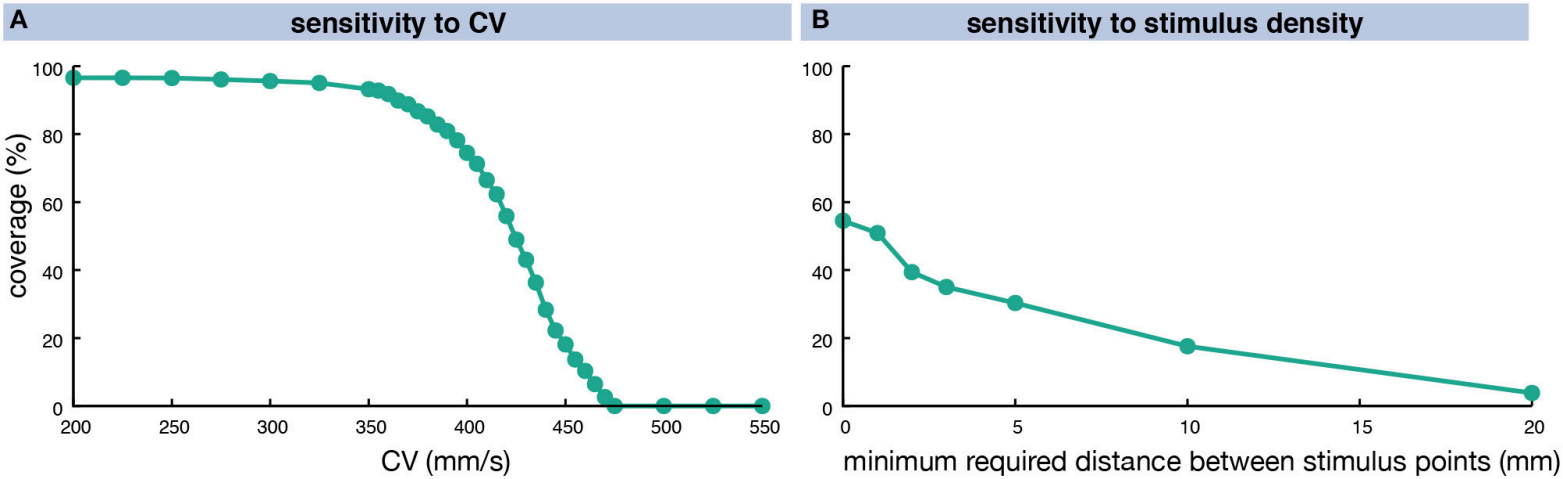
Sensitivity of vulnerable pathway coverage to changes in homogeneous, anisotropic CV **(A)** and the stimulus point density **(B)**. The CV was altered in a homogeneous, anisotropic setup causing different degrees of RA coverage by vulnerable pathways for a fixed stimulus density of 1 mm. In **(B)**, the distance between stimulus points was varied for a fixed CV of 425 mm/s.

The number of vulnerable pathways and the share of RA myocardium covered by them was highly dependent on the CV. In a homogeneous anisotropic setup (ERP 318 ms at long BCLs), the coverage increased from 0% at a CV of 475 mm/s to over 90% for CVs of 360 mm/s and lower (Figure 7A). When interpreting these findings, one should keep in mind the homogeneous ERP restitution used in this experiment (solid green line in Figure 3B). The degree of coverage also depends on the number of different stimulus locations evaluated (Figure 7B). Considering all 19,296 RA nodes yielded a coverage of 54.5% for a fixed CV of 425 mm/s. Requiring a minimum distance of 1 mm between stimulus points reduced their number to 8,254 without affecting the coverage result markedly (50.9%). Considering less points yielded lower coverage rates (39.4% for 2 mm $\hat{=}$ 2,136 nodes, 3.8% for 20 mm $\hat{=}$ 19 nodes).

The degree of coverage was highly dependent on the substrate as detailed in Table 1. 8,254 stimulus points with a minimum distance of 1 mm were considered using the CV and ERP values fitted from the monodomain model output using the biophysically detailed cell models given in Supplementary Table 1. While the fitted exponential restitution of ERP and CV was modeled homogeneously across the RA, its region-dependent heterogeneous anisotropy (*k* in Supplementary Table 1) was kept. To operate in an interesting range of vulnerability (Figure 7A), CV at long BCLs (*A* in Supplementary Table 1) was set to 454 mm/s in the following yielding a total RA activation time of 194 ms in the *control* model without any drug applied.

**Table 1:** Degree of coverage of RA elements with pathways vulnerable to AFlut for different substrates and antiarrhythmic drugs amiodarone (*amio*) and dronedarone (*drone*).

	**Degree of coverage**					
	**Original CV**			**Total activation time matched**		
	**no drug**	**amio**	**drone**	**no drug**	**amio**	**drone**
Control	18.1%	70.5%	0.0%	18.1%	0.0%	0.0%
cAF	96.0%	96.3%	95.6%	96.2%	96.1%	94.9%
N588K	93.2%	94.1%	0.0%	93.0%	77.8%	0.0%
L532P	96.2%	96.5%	34.5%	96.3%	96.0%	11.1%

*In the right three columns, the A and B values determining the CV according to Equation (3) were scaled to match the activation time of the last element during sinus rhythm in the control substrate with no drug applied*.

**Figure 8:**
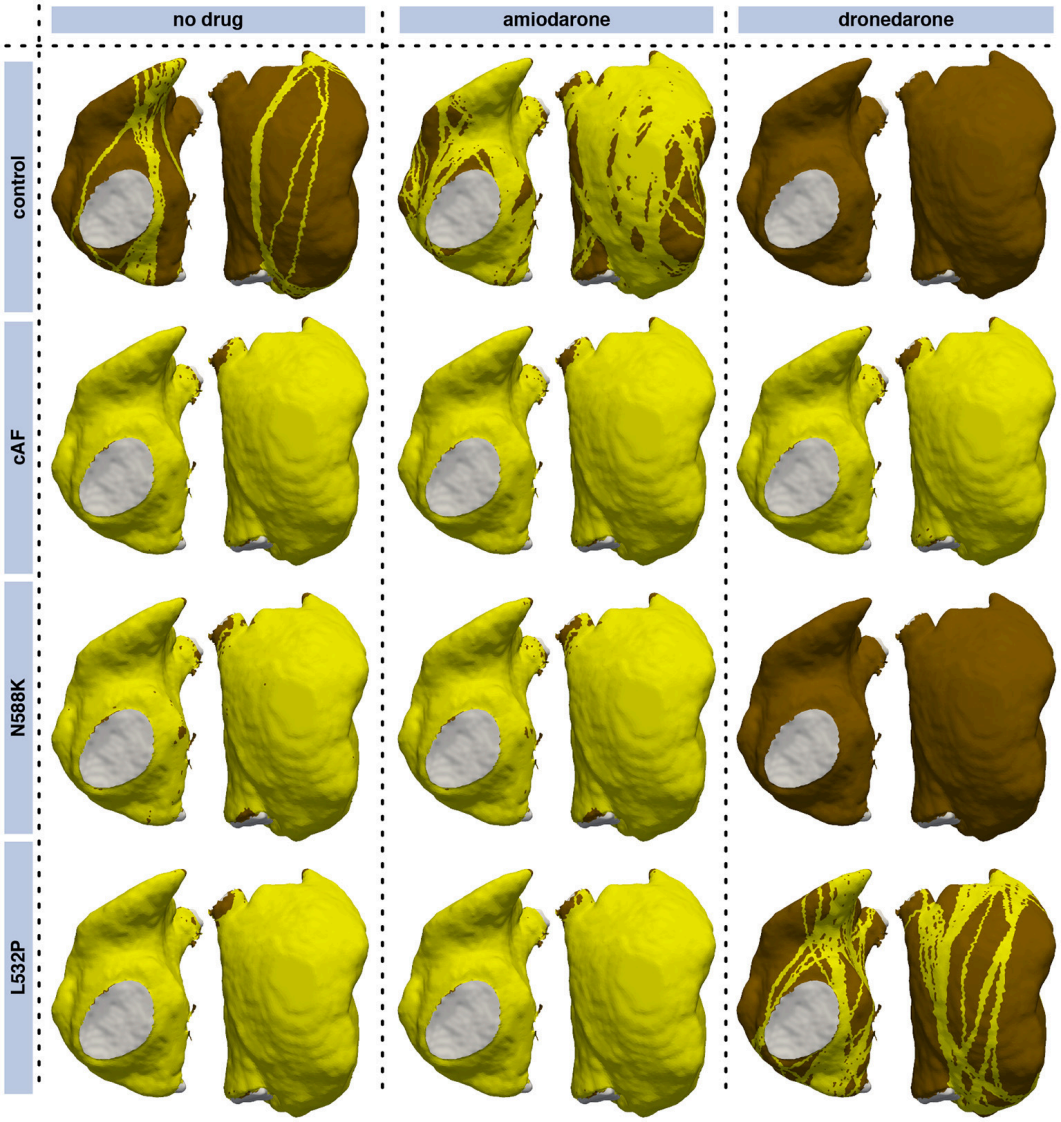
Vulnerability maps of the RA for combinations of different substrates and pharmacological agents. Besides a control substrate representing healthy myocytes, a cAF remodeled substrate (neglecting changes of cell-to-cell coupling), and the two hERG mutations N588K and L532P were evaluated. Standard concentrations of the antiarrhythmic agents amiodarone (2.3 μM) and dronedarone (0.21 μM) were administered in the center and right columns, respectively. Vulnerable pathways are marked in yellow on the RA myocardium (brown); the blood pool is indicated in gray. Supplementary Figure 4 shows the results for a matched total sinus rhythm activation time in all setups.

Both the cAF substrate and the two hERG mutations were more vulnerable to AFlut than the control model representing healthy myocytes. The higher degree of coverage under the influence of amiodarone observed for all substrates can be explained by the WL restitution (Figure 3F). The WL was shortened by the administration of amiodarone due to conduction slowing caused by the sodium channel inhibition. This effect was most pronounced in the control substrate and was reflected in the vulnerability maps as well (Figure 8).

In order to separate the effects of the different substrates and compounds on CV and ERP, the total activation time of the RA was matched with the activation of the last element in the control model and no drug (192 ms) in a second set of simulations (Supplementary Figure 4), i.e., the *A* and *B* parameters determining the CV according to Equation (3) were scaled while keeping the anisotropy ratio *k* constant. In this way, only the effect on repolarization (ERP) was considered leading to a reduction of vulnerable pathways under the influence of amiodarone in all substrates and a more pronounced reduction under the influence of dronedarone compared to Figure 8.

**Figure 9:**
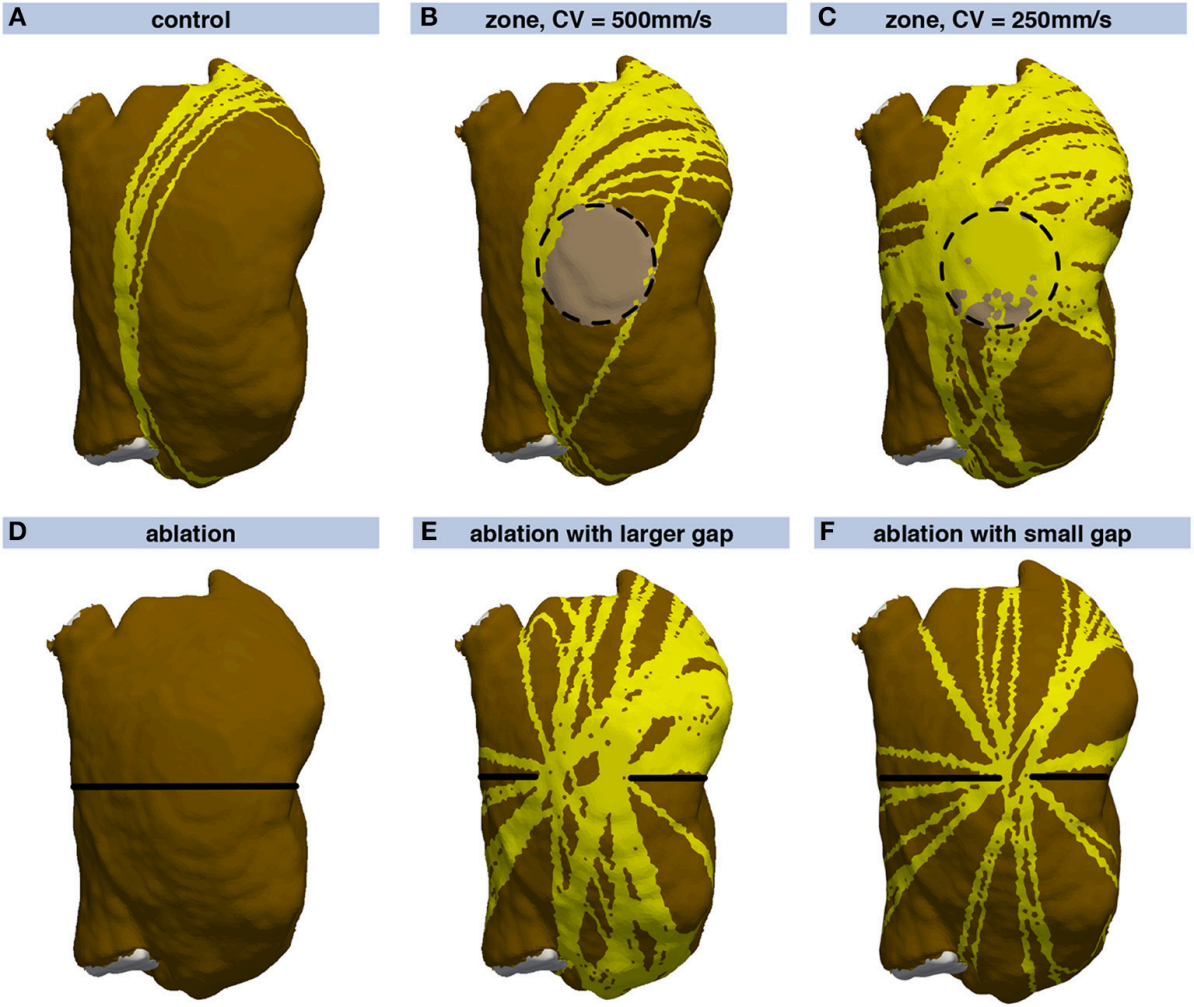
Vulnerability maps of the RA for different substrate modifications. Compared, to **(A)**, a zone of slow conduction was introduced in **(B,C)** (light brown area enclosed by dashed circle). A circular ablation lesion (black line) was introduced in **(D)**. In **(E,F)**, a gap in the ablation lesion with varying extent was modeled on the central posterior wall.

Besides evaluating different substrates, distinct spatial heterogeneities were introduced in the RA model. The normal RA myocardium was parametrized with an isotropic CV of 700 mm/s and an ERP of 250 ms for all BCLs. A circular zone of slow conduction on the posterior wall was modeled (Figures 9B,C). Depending on the CVs inside and outside this zone, the wave might be faster bypassing the zone than propagating through it. Comparing the time the wave takes to bypass the circle with the time it takes to propagate through the zone of slow conduction yields a critical CV_*slow*_/CV_*normal*_ ratio of 2/π≈0.63. If the ratio is higher, the dominant pathway is through the zone of slow conduction. If it is lower, the bypassing wave is faster. Therefore, the zone of slow conduction within the surrounding tissue conducting at 700 mm/s was parametrized with a CV of 500 mm/s resulting in a ratio of 0.71 (Figure 9B), and 250 mm/s ($\hat{=}$ 0.36, Figure 9C). In contrast to the control model (Figure 9A), the zones of slow conduction yielded additional flutter pathways. For the CV of 500 mm/s in the zone of slow conduction, 24.1% of the RA were covered by vulnerable pathways (Figure 9B) in contrast to 14.1% in the control case (Figure 9A). Additional flutter pathways crossed the periphery of the zone of slow conduction and thereby prolonged the RTT. For the slower CV of 250 mm/s, the entire zone of slow conduction was covered by vulnerable pathways yielding a total RA coverage of 47.8% (Figure 9C). The pathways were not constricted to the faster route outside the zone as the route through the zone of slow conduction was optimal considering the field of view of the geometrical snake. When computing an inducability map (see Discussion), the driving pathway would be running around the zone of slow conduction, though.

The second spatial substrate modification was an ablation lesion which encircled the RA completely (Figure 9D). Rather than being a clinically used ablation pattern, this scenario serves as an example separating the RA into two electrically isolated regions. The lesion was modeled as non-conductive, thus no flutter pathways could cross it. In additional scenarios, a gap in the ablation lesion of varying extent was assumed at the central posterior wall (Figures 9E,F). In case of the complete lesion, no flutter pathways were identified (Figure 9D) as the WL condition could not be fulfilled on any of the two separated, smaller substrates. The gap in the ablation lesion yielded numerous vulnerable pathways running through the gap at various angles (Figure 9E). The flutter pathways covered 42.9% of the RA in contrast to 14.1% in the control case, thus the ablation increased AFlut vulnerability markedly. In case of a smaller gap (Figure 9F), the number of loops was smaller than for the wider gap as the narrow gap served as a funnel. Nevertheless, the coverage (23.3%) was still markedly higher than for the control case.

The time to compute a complete vulnerability map depends on the mesh resolution as well as the number of stimulus points considered and the number of loop candidates fulfilling the WL condition over time. The computation is faster, the fewer loop candidates there are and the earlier the constricted loops are disregarded because they no longer fulfill the WL condition (data not shown). For the RA mesh consisting of 19,296 nodes, computation was timed on an *Intel Xeon E5-2697V2* machine with twelve cores at a base clock rate of 2.7 GHz. The control vulnerability map in Figure 9A with a coverage of 14.1% was computed within 4.0 min whereas it took 5.5 min to compute the vulnerability map for the RA including the zone of slow conduction yielding a coverage of 47.8% (Figure 9C).

### 3.3. Phase Extrapolation

The vulnerable pathways represented in the vulnerability maps and identified using the methods described above were extrapolated on the whole RA in terms of phase using the methods described in section 2.4. Each vulnerable pathway was extrapolated in phase space individually. The eikonal-diffusion approach converged within 16 to 18 iterations and was robust against variations of the CV [Supplementary Equation (7)] and the RTT [Supplementary Equation (9)], i.e., the cycle length of the reentry. CV was varied between 0.1 × and 2 × the ground truth value used in the fast marching simulation and assumed RTTs between 0.3 × and 3 × the ground truth value. The phase map was then used to determine the initial state of a dynamic fast marching simulation as exemplified in the next section for a clinical dataset.

### 3.4. Clinical Example

**Figure 10:**
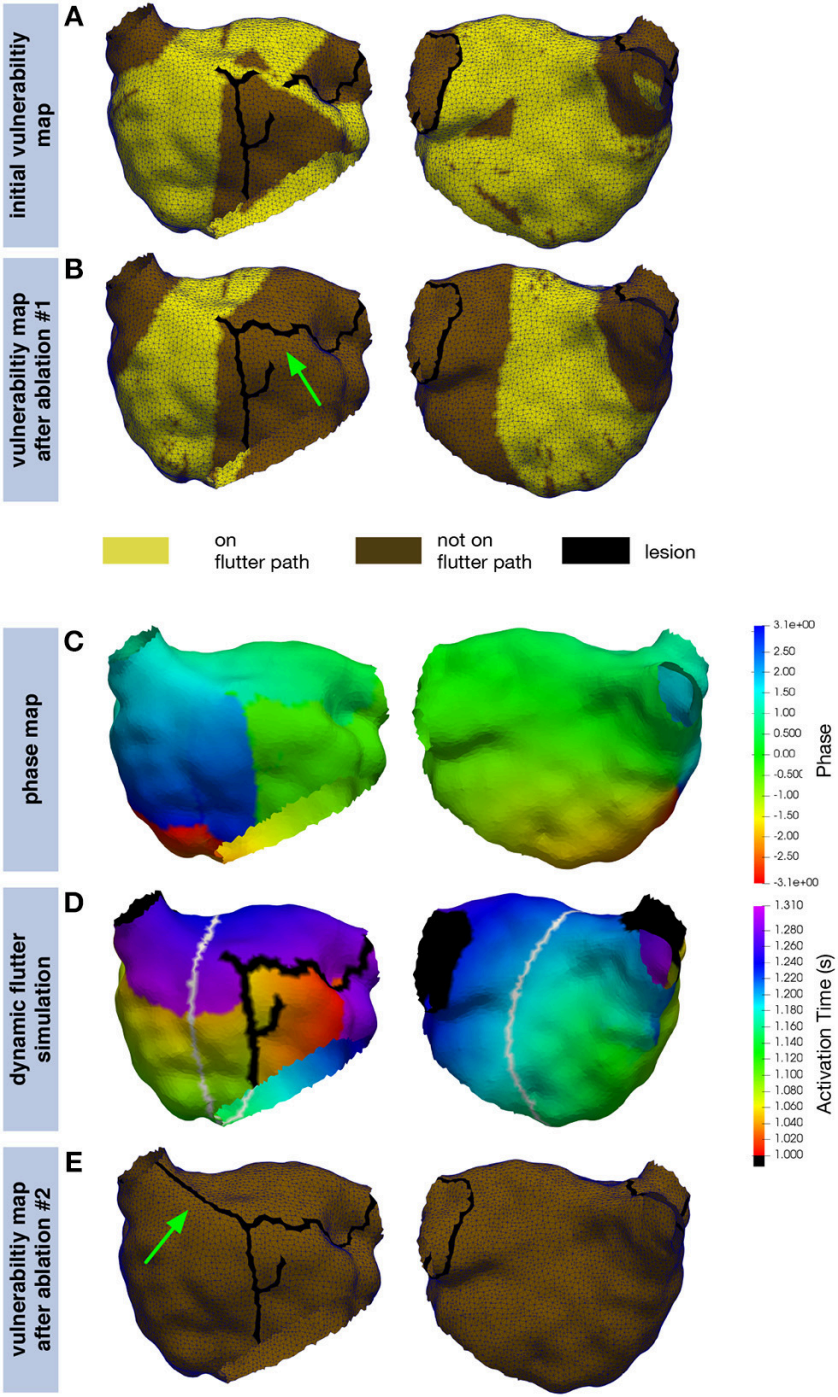
Application of the proposed simulation pipeline on a clinical example. The inital vulnerability map **(A)** shows numerous pathways along which AFlut can be sustained. By closing the gap in the previous line on the left part of the anterior wall (green arrow), the number of pathways could be reduced but a significant amount remained **(B)**. The extrapolated phase map **(C)** for one of the pathways identified in **(B)** (gray line in **D**) was used to intialize a dynamic fast marching simulation **(D)** exhibiting stable reentry. Additional ablation to the (RSPV) (green arrow) eliminated all vulnerable pathways **(E)**.

The inital vulnerability map representing the state with which the patient presented for the redo procedure (compare section 2.6) covered most parts of the LA (Figure 10A). AFlut pathways running through the gap in the anterior line were present. After closing this gap, the left part of the anterior wall became free from AFlut pathways and they were restricted to the central part of the posterior wall (Figure 10B). However, there were still numerous AFlut pathways remaining. These pathways (the gray line in Figure 10D shows one example) were used to extrapolate a phase map on the whole domain (Figure 10C) and intialize a dynamic fast marching flutter simulation (Figure 10D). Reentry was stable for the entire simulation duration of 100 reentrant cycles. After connecting the line on the anterior wall to the RSPV by additional ablation, no more vulnerable pathways could be identified (Figure 10E).

## 4. Discussion

### 4.1. Main Findings

In this study, a simulation workflow to identify vulnerable pathways potentially sustaining AFlut was presented. The approach builds on personalized fast marching simulations of excitation propagation and geometric snakes to constrict pathways identified on the basis of wavefront collision sites. Throughout the whole pipeline, heterogeneous, anisotropic, and heart rate-dependent tissue properties are considered in terms of CV and ERP.

The geometrical snake approach presented by Bischoff and Kobbelt (2004) was adapted to the excitation propagation application scenario considering the tissue property heterogeneities mentioned above. Applying the geometric snake approach to loop candidates identified as circular pathways from an initial stimulus point via a site of wavefront collision back to the initial stimulus yielded AFlut vulnerability maps. The number and the location of the identified vulnerable flutter pathways were sensitive to (i) anisotropy (Figure 6), (ii) the substrate properties regarding CV and repolarization, as well as modification of these parameters due to pharmacological compounds (Figure 8 and Supplementary Figure 4), (iii) zones of slow conduction or ablation lesions (Figure 9), and most importantly (iv) the assumed CV (Figure 7). Anisotropic substrates were more vulnerable than isotropic tissue when matching the total sinus rhythm activation time of the RA. The WL is a crucial parameter as can be seen by the higher number of vulnerable pathways identified for the cAF and hERG mutated substrates compared to control. While dronedarone reduced the AFlut vulnerability, amiodarone rendered the substrate more vulnerable due to the reduced WL caused by the slowed CV (Figure 3). When only considering the effect on repolarization, thus altering ERP to represent the influence of the drug, amiodarone exhibited antiarrhythmic properties as well. The effect was less pronounced than for dronedarone, though. Zones of slow conduction increased the number and the density of vulnerable pathways as the cycle length increases both by conducting through the slow zone and by bypassing it. Ablation lesions isolating different regions completely rendered the RA invulnerable to AFlut. However, even a small gap in the lesion increased the number of vulnerable pathways markedly compared to control. This effect can be explained by the narrow isthmus formed by the gap in the lesion and highlights the importance of lesion durability. When linear lesions are partly revitalizing, they form ideal pathways for AFlut. In particular, shortcuts leading to wavefront collision and ceasing the reentrant activation are cut off by the lesion, thus stabilizing the reentry. The assumed CV had the biggest effect on the number of vulnerable pathways and the degree of RA coverage by them. A CV slowing by 25% rendered an invulnerable RA model highly vulnerable with a flutter pathway coverage of over 90%. This finding highlights the importance of a reliable CV estimation to draw relevant conclusions from personalized models using the method presented here. The CV of the individual patient has to be measured in a spatially resolved, and preferably heart rate-dependent, manner. Weber et al. (2010, 2011) proposed a method to estimate local CV and its restitution based on a cosine fit method. The advent of new electro-anatomical mapping systems and catheters with improved signal quality as well as sophisticated signal processing approaches gives rise to hope for such CV mapping in the near future (Latcu and Saoudi, 2014; Cantwell et al., 2015; Verma et al., 2018).

The application to a clinical example can be considered a proof of concept. We built on the individual anatomy exported from the mapping system and tailored the substrate to the clinically observed activation pattern. The model prediction and the clinical observations show good qualitative agreement. However, it has to be considered that the substrate was modeled homogeneously apart from the lesions and restitution properties were not taken into account. Therefore the lack of exact quantitative match is not surprising.

The computation of a single activation sequence was faster than real-time, a complete vulnerability map took several minutes. Most of the computational cost was due to the constriction of the loop candidates using the geometrical snake approach. While the time spent to calculate excitation propagation accounted for only a minor share, less complex alternatives to the fast marching algorithm exist. Graph-based approaches, such as the A^*^ algorithm (Hart et al., 1968; Wallman et al., 2012) or the fastest route algorithm (van Dam et al., 2009) are however only faster if the activation time at only a subset of nodes is needed. Cellular automata on the other hand do not consider quadratic approximation of activation times. The computational complexity of the geometrical snake implementation could be reduced by optimizing the number of neighbors considered for the calculation of the snaxel velocity [Supplementary Equation (4)] and the convergence criteria. Indeed, the approach considering *N* = 30 neighbors with decreasing weight could be approximated by a spatial multi-grid approach starting with distant neighbors in early iterations and focussing on closer nodes at later iterations. When aiming at an interactive modification of the substrate, e.g., by introducing virtual ablation lesions, results from previous evaluations can be reused for regions not affected by the last modification. Moreover, intermediate results could be precomputed, thus trading memory footprint in for reduced computation time. This potential for optimizations together with parallel computing approaches make interactive assessment of ablation therapy in almost real-time appear achievable.

### 4.2. Relation to Previous Work

Dang et al. (2005) and Reumann et al. (2008) compared different standard ablation patterns for AF using idealized computational models and suggest that PV isolation together with linear lesions is the most effective treatment for AF. However, they did not investigate the vulnerability to AFlut post AF ablation systematically. Hwang et al. (2014) proposed a method to test AF ablation patterns *in silico* using a monodomain approach on anatomically, but not electrophysiologically personalized models. Thus, no substrate information regarding fibrosis, zones of slow conduction or the degree of electrophysiological remodeling is considered. Recently, Alessandrini et al. (2018) presented an *in silico* framework to holistically consider the entire cycle from excitation propagation, catheter deformation, electrogram acquisition and processing to virtual ablation. McDowell et al. (2015) published a proof-of-concept how computational modeling can predict ablation sites terminating rotors driving AF in personalized models including fibrosis distribution. Bayer et al. (2016) evaluated the potency of PV isolation, mitral and roof lines, ablation guided by rotor mapping, and lesions streamlining sinus activation regarding the termination of AF *in silico*. The potential of clinically-derived computational models to optimize catheter ablation of AF was recently reviewed by Zhao et al. (2015). They conclude that high-resolution three-dimensional models of functionally and structurally mapped atria of the exact patient are imperative to provide clinically relevant insights on a personalized level.

Lines et al. (2009) presented a method to parametrize a monodomain simulation in a standard bi-atrial model aiming to incorporate electrograms acquired during electroanatomic mapping studies in order to replicate clinically mapped AFlut *in silico*. The extracellular potentials at 32 computational nodes served as a boundary condition for the solution of the monodomain system. While the algorithm synchronized the simulation to the synthetic reference simulation, the algorithm is computationally expensive and only allows to study clinically observed cases *in silico* but cannot provide information on the vulnerability to AFlut.

While those previous studies assessed ablation patterns regarding the prevention or termination of AF, this is the first work to assess the vulnerability to AFlut based on an individualized anatomical model besides a recent work by Zahid et al. (2016) to the best of our knowledge (see also reviews by Jacquemet, 2016; Boyle et al., 2017). Zahid et al. employed the minimum cut algorithm to predict optimal ablation sites for AFlut in the LA with substantially increased computational effort. Child et al. (2015) introduced the reentry vulnerability index (RVI) as a quantitative metric based on the difference between activation and repolarization intervals at pairs of spatial locations. The RVI correlates with exit sites of scar-related reentrant arrhythmia as commonly observed in the ventricles (Hill et al., 2016). However, it aims at predicting functional lines of block rather than providing a comprehensive map of vulnerable pathways based on the individual geometrical properties. The same holds for a study by Wallman et al. (2013) quantifying the arrhythmogeneity of scar and left-to-right heterogeneity in the ventricles.

Trächtler et al. (2015) used the fast marching implementation presented here for a similar *in silico* reproduction of clinical cases. While both methods allow to test ablation strategies regarding the termination of the specific reentry, they do not allow to draw conclusions regarding the vulnerability to AFlut along other pathways. Thus, these approaches do not provide the means to optimize AF ablation aiming at the prevention of post-ablational AFlut.

### 4.3. Outlook

The method presented here could be further developed regarding two aspects. First, the extrapolated phase map obtained by the eikonal-diffusion approach could not only be used to initialize a fast marching simulation but also to replicate the flutter pathway in a monodomain simulation. Matene and Jacquemet (2012) proposed a suitable approach, which they used to initiate AF by extrapolating phase singularities (Jacquemet, 2010; Herlin and Jacquemet, 2011). By initiating the same flutter loop in both the fast marching and the monodomain environment, the fast marching approach could be further validated with respect to macro-reentry. Second, the dominant flutter pathways sustaining reentry in the dynamic simulations could be tracked and compared to the pathways used to extrapolate the initial state. In this way, not only a map of vulnerable flutter pathways but also a map of inducible flutter pathways could be computed.

Moving forward, the complete simulation pipeline should be validated in the clinical setting once tools for a spatially resolved CV and ERP estimation become available. The anatomical model of the individual patient built from MRI data could be augmented with a priori knowledge (Wachter et al., 2015). CV and ERP would be parametrized using intracardiac measurements (Unger et al., 2017; Verma et al., 2018) and complemented with rule-based assumptions. Preferably, the subjects should be recruited from patients undergoing ablation of AFlut that developed after AF ablation. Ideal validation cases would be formed by patients in which a gap in the intial ablation lesion set is identified during the second procedure. The lesions placed during the initial AF ablation procedure as well as the gap in it would be included in the suggested pipeline as further a priori knowledge. The clinically observed flutter pathway should then be found in the vulnerability and inducability map. Moreover, the ablation terminating the flutter in the clinical setting should also remove the specific vulnerable pathway from the map. In this way, the concepts and methodologies for both vulnerabiltiy and inducability maps could be clinically validated.

### 4.4. Limitations

The implementation of the fast marching algorithm used in this work does consider anisotropic CV but does not consider recursive anisotropic correction as proposed by (Sermesant et al., 2007). In Pernod et al. (2011), the authors of Sermesant et al. (2007) show that the computation time is higher by a factor of ≈1.6 when considering anisoptropic correction. While the influence of the anisotropic correction has never been evaluated systematically, it should not be too relevant for moderate anisotropy values. For the application presented in this work, subtle differences of the activation sequence do not play a major role for the final result as fast marching activation times serve only as the input for subsequent processing.

Another limitation of the presented method is its restriction to monoatrial flutter pathways. The reason for this can be seen in Supplementary Figure 5 in which a biatrial loop candidate was constricted using the geometrical snakes approach. While a shortcut within the LA exists, it cannot be resolved by the snake as it is constrained by the interatrial connections and can thus not cross the septum. However, the method could be extended to identify shortcuts within the two atria by also considering monoatrial loops in addition. The final constricted biatrial loop could be used to initialize additional monoatrial loops comprising the segment of the biatrial loop and shortest connection between the two open ends at the interatrial connections. When computing inducability maps instead of vulnerability maps, this limitation is not relevant since reentry along a loop as in Supplementary Figure 5 could not be induced if this would not be the case for a comparable monoatrial loop as well. Regarding the dynamic simulation of AFlut, the missing representation of electrotonic coupling is a limitation.

The biggest hurdle is the sparsity of the available clinical data to characterize an individual's substrate and the associated uncertainty. The importance of a reliable CV estimation is highlighted by the fact that a CV uncertainty of Δ*c* corresponds to scaling of the atrium by a factor of $\sqrt{\Delta c}$ (Jacquemet, 2016). Considering that the minimal WL needed to sustain reentry is defined by the product of CV and ERP, uncertainty of ERP plays an important role as well (Jacquemet et al., 2005; Krogh-Madsen and Christini, 2012; Deng et al., 2017). Improved electro-anatomic mapping systems providing better signal quality and simultaneous mapping using a multitude of electrodes, as well as advanced signal processing methods (Cantwell et al., 2015; Unger et al., 2017; Verma et al., 2018) make it seem probable to have suitable data available in the near future. Moreover, the uncertainty in the data could be taken into account by probabilistic modeling using Bayesian inference and compressed sensing methods (Konukoglu et al., 2011).

### 4.5. Conclusion

We presented a comprehensive method to analyze the vulnerability to AFlut in a personalized way and demonstrated its applicability for clinical data. The individual anatomy as well as electrophysiology in terms of CV, ERP, and their heart rate dependence is taken into account. This tool provides the means to evaluate potential ablation strategies *in silico* regarding their arrhythmic potential for AFlut before actually applying them in the electrophysiology lab. In this way, this work can be one piece in the puzzle to overcome the *learning by burning* paradigm and eventually reduce the number of patients suffering from post-ablational AFlut.

## Disclosure

Part of this study was published before as a thesis (Loewe, 2016).

## Author Contributions

ALo, TO, GS, and OD concepted the study. ALo, EP, and TO implemented the algorithms and performed the *in silico* experiments. ALo, EP, TO, GS, and OD analyzed and interpreted the data. ALu and CS performed the clinical procedure. ALo drafted the manuscript. EP, TO, ALu, CS, GS, and OD revised the manuscript critically. All authors approved the final version of the manuscript.

### Conflict of Interest Statement

The authors declare that the research was conducted in the absence of any commercial or financial relationships that could be construed as a potential conflict of interest.
