# Supplementary material for: Patient-Specific Identification of Atrial Flutter Vulnerability–A Computational Approach to Reveal Latent Reentry Pathways
Source: Front Physiol. 2019 Jan 14;9:1910. doi: 10.3389/fphys.2018.01910 (PMC6339942; doi:10.3389/fphys.2018.01910)
Supplement: Supplementary file 1 [file Data_Sheet_1.PDF]

frontiers

Supplementary Material: Patient-Specific Identification of Atrial Flutter Vulnerability - A Computational Approach to Reveal Latent Reentry Pathways

Axel Loewe [1,*], Emanuel Poremba [1], Tobias Oesterlein [1], Armin Luik [2], Claus Schmitt [2], Gunnar Seemann [1,3,4], and Olaf Dössel [1]
*Correspondence:
Axel Loewe
publications@ibt.kit.edu

1 SUPPLEMENTARY METHODS

1.1 Fast Marching Simulation of Excitation Propagation

1.2 Constriction of Flutter Loop Candidates

The geometric snake is an active contour model that is restricted to a polygonal surface mesh. In this work, the parametrization-free implementation for triangular meshes proposed by Bischoff and Kobbelt (2004) was used. A snake is represented as a polygon in space (Supplementary Figure 2). The vertices of the snake are referred to as *snaxels* and represented by lower case vectors in the following. Snaxels are constrained to lie on edges of the mesh. Furthermore, the segments of the snake (connections between snaxels) have to lie in the interior of triangles. An oriented snaxel $\vec{s}$ can thus be defined as:

$$\vec{s} = \vec{f_s} + p_s \cdot (\vec{t_s} - \vec{f_s}) \; , \tag{1}$$

with the points $\vec{t_s}$ and $\vec{f_s}$ defining the supporting edge on which the snaxel $\vec{s}$ lies and $p_s \in (0,1)$ defining the position on the edge.
The snake evolves by assigning a scalar speed v_s to each snaxel $\vec{s}$:

$$p_s \leftarrow p_s + \Delta t \cdot v_s \; , \tag{2}$$

with Δt being a virtual time increment. The time increment was chosen as 0.5 in this study striking a good balance between stability and convergence speed. By performing the update step, the snaxel is shifted along the supporting edge of the mesh $\vec{e_s}$ (Supplementary Figure 3):

$$\vec{e_s} = \vec{t_s} - \vec{f_s} \; . \tag{3}$$

In order to avoid local oscillations, not only the direct neighbors were considered when calculating the velocity:

$$v_s = \frac{1}{\left(\frac{\sqrt{\vec{e_s} \cdot \vec{H_s} \cdot \vec{e_s}}}{CV_S(RTT)}\right) \cdot \sum_{i=0}^{N-1} a^i} \cdot \sum_{i=0}^{N-1} \left[a^i \left(d\left(\vec{p_i}, \vec{s}\right) + d\left(\vec{n_i}, \vec{s}\right)\right)\right] \; , \tag{4}$$

Algorithm 1 Multifront fast marching. In each iteration, the *UNKNOWN* neighbors of nodes in the *KNOWN* list (initially populated with the excitation origin) are added to the *TRIAL* list. The node with the smallest t_a in *TRIAL* is moved to *KNOWN* and t_a is updated for the remaining *TRIAL* nodes. After the activity period (AcP) has passed, *KNOWN* nodes are moved to the *REFRACTORY* list from which they are moved to the *UNKNOWN* list after the ERP has passed. The algorithm terminates if the *TRIAL* list becomes empty or the integrated time (IT) exceeds the predefined simulation time.

```
IT ← 0.0
while (TRIAL ≠ ∅) ∧ (IT < maxTime) do
    ET ← 0.0
    while (TRIAL ≠ ∅) ∧ (ET < timeStep) do
        X ← arg min_{X∈TRIAL} {t_a (X)}
        TRIAL ← TRIAL \ {X}
        KNOWN ← KNOWN ∪ {X}
        for all (X_i ∈ N (X)) ∧ (X_i ∈ UNKNOWN) do
            t_a (X_i) ← update(X_i, X)
            if X_i ∉ TRIAL then
                TRIAL ← TRIAL ∪ {X_i}
            end if
            ET ← t_a (X_i) − IT
        end for
    end while
    IT ← IT + timeStep
    for all X ∈ KNOWN do
        if (IT − t_a (X)) > AcP (X) then
            KNOWN ← KNOWN \ {X}
            REFRACTORY ← REFRACTORY ∪ {X}
        end if
    end for
    for all X ∈ REFRACTORY do
        if (IT − t_a(X)) > ERP(X) then
            REFRACTORY ← REFRACTORY \ {X}
            UNKNOWN ← UNKNOWN ∪ {X}
        end if
    end for
end while
```

with N being the order of the approach (i.e., the number of predecessor and successor snaxels considered), a being the order divisor $\in$ (0,1] leading to a higher weight for closer snaxels, and $\vec{H}$ being the distance anisotropy tensor reciprocal to $\vec{G}$ in Equation (1) of the main manuscript:

$$\vec{H} = R(\phi, \theta) \begin{pmatrix} k^{-1} & 0 & 0 \\ 0 & 1 & 0 \\ 0 & 0 & 1 \end{pmatrix} R(\phi, \theta)^T \ , \tag{5}$$

As snaxels are located on edges between two nodes and the material properties conduction velocity (CV) and effective refractory period (ERP) are defined for each node, the closer node was considered (referred to by upper case vectors using the same letter). Thus, $CV_S(RTT)$ is the CV of the closest node to snaxel $\vec{s}$ considering the round trip time (RTT) of the last iteration as basic cycle length (BCL) according to Equation (2) in the main manuscript. p_i is the ith predecessor of snaxel s and n_i is the ith successor. In this study, the order N was chosen as 30 and a was set to 0.9 based on experience gained in pilot studies. The

anisotropic distance projected on the supporting edge and weighted by the heterogeneous CV is defined as:

$$d\left(\vec{p_1}, \vec{p_2}\right) = \frac{1}{CV_{P_1}(RTT)} \left(\vec{p_1} - \vec{p_2}\right) \vec{H_{P_1}} \cdot \frac{\vec{e_s}}{\|\vec{e_s}\|_2} \ , \tag{6}$$

If an update step shifts a snaxel onto one of the supporting nodes or beyond them ($p_s \leq 0$ or $p_s \geq 1$), the snaxel is duplicated and distributed on all adjacent edges with an initial p_s of 0.05. Thus, the new snaxels are located 5% away from the node being crossed with respect to the length of their new supporting edge. During the evolution, one more constraint is checked and enforced: no two consecutive snake segments may lie within the same triangle. If this was violated because a snaxel was distributed after passing a supporting node, the interior snaxel was disregarded and the first and third snaxel were connected directly. Snaxel collisions caused by snaxels crossing each other on the same supporting edge are resolved by merging the respective snake segments. After each iteration, the wavelength condition (Equation (4) in the main manuscript) is checked. Snakes not fulfilling it are disregarded immediately. The iterative algorithm was stopped once the absolute RTT reduction over the last 20 iterations was less than 7 ms or the relative reduction was less than 10%. This choice of parameters yielded stable convergence and is further discussed below.

The chosen approach controls the topology, detects and resolves self-collisions at sub-element size precision, and inherently avoids error prone back projections of snaxels onto the mesh.

1.3 Eikonal-Diffusion Phase Extrapolation

Jacquemet proposed an eikonal-diffusion approach for the initiation of reentrant cardiac propagation (Jacquemet, 2010, 2012). The eikonal-diffusion equation can be derived from the monodomain equation using singular perturbation theory (Jacquemet, 2010; Tomlinson et al., 2002; Colli Franzone et al., 1990):

$$\|\vec{c}\nabla t_a\|_2 = 1 + \nabla \cdot \left(\vec{D}\nabla t_a\right) \qquad x \in \Omega \ , \tag{7}$$

$$\vec{n} \cdot \vec{D}\nabla t_a = 0 \qquad x \in \partial\Omega \ , \tag{8}$$

with the symmetric positive definite tensors $\vec{c}$ and $\vec{D}$ being the link to the monodomain equation, Ω being the computation domain, and $\vec{n}$ being the unit vector normal to the boundary $\partial\Omega$.

We transform the activation times t_a of the nodes along the flutter loop to phase space:

$$\phi\left(x_i\right) = 2\pi\frac{t_a\left(x_i\right)}{RTT} \ . \tag{9}$$

Laplacian interpolation of the input phase distribution (potentially including phase singularities) is used to obtain an initial estimate of the phase distribution $\phi(x)$ on the whole domain. Then, a linearized eikonal approach is used to iteratively correct the phase distribution to satisfy Equation (1) in the main manuscript (Jacquemet, 2012). The resulting phase on the whole computational domain can be used to initialize a dynamic fast marching simulation. The nodes in the first 50% of the cycle are included in the *REFRACTORY* list, the next 20% in *KNOWN*, the next 20% are included in the *TRIAL* list, and the last 10% in *UNKNOWN*. Nodes in the *KNOWN* list have a fixed activation time influencing the activation time of the nodes in the *TRIAL* list, i.e., the *KNOWN* list holds the nodes of the activation wavefront. The element with the smallest timestamp in the *TRIAL* list (holding the nodes soon to be activated) is added to the *KNOWN* list and all *UNKNOWN*, i.e., excitable neighbors are added to the trial list (Supplementary Algorithm 1). Thus, the simulation is initialized with a temporal excitable gap of 10% of the RTT (*UNKNOWN*). The BCL of all

nodes was initialized with the RTT and the time of the last activation t_a of each node i was set by mapping the phase back to an activation time and starting the dynamic simulation at time $t = RTT$:

$$t_a(x_i) = RTT \frac{\phi(x_i)}{2\pi} \; . \qquad (10)$$

2 SUPPLEMENTARY FIGURES

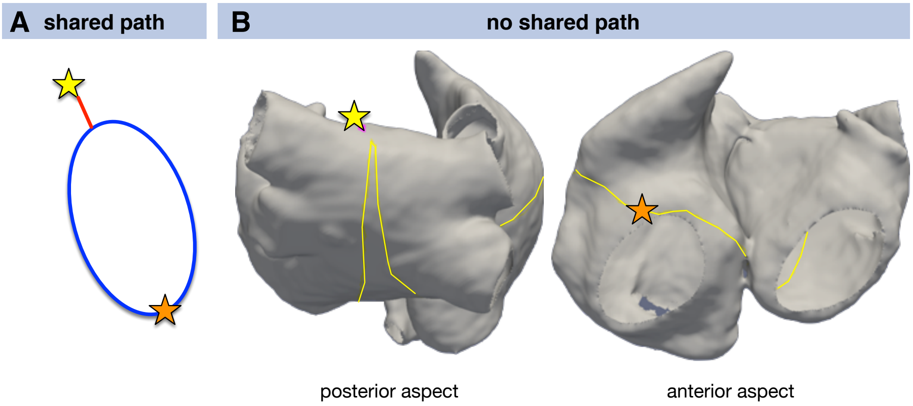

Figure 1: Artifacts introduced by loop tracing from sites of wavefront collision (orange star) to the stimulus location (yellow star). As in (A) both half loops share part of the loop (red segment), the loop can be constricted to the blue circle. In (B), the wavefronts collided on the anterior wall and the shared pathway was already disregarded (note the distance between the yellow star and the yellow loop on the left atrial roof). However, the loop would still be cut short by a propagating wave between the posterior interatrial connections and the conneciton at the coronary sinus.

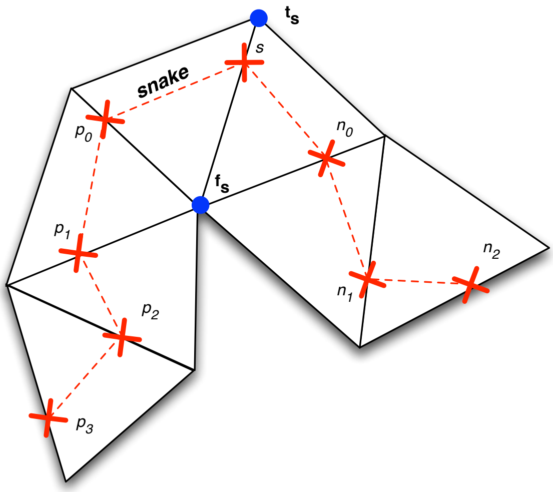

Figure 2: Geometric snake on a triangular mesh. The snake segments run on the surface of the triangles from snaxel to snaxel. Each snaxel is constrained to its supporting edge. Snaxel $\vec{s}$ is supported by the edge running from $\vec{f_s}$ to $\vec{t_s}$ and directly connected to its predecessor $\vec{p_0}$ and its successor $\vec{n_0}$.

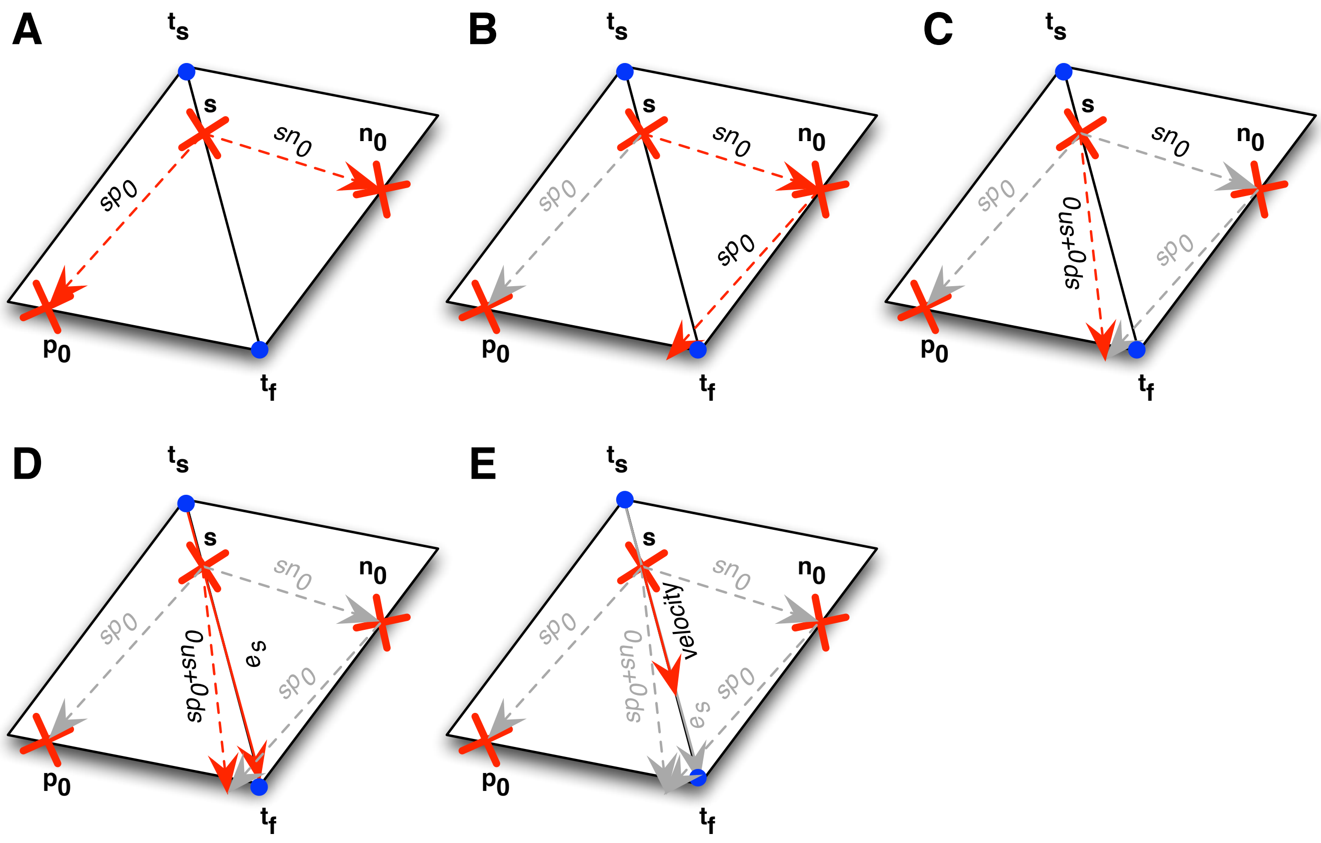

Figure 3: Concept of the snaxel velocity calculation for order N=1 and isotropic tissue properties. Snaxel $\vec{s}$ is connected to its first order neighbors $\vec{p_0}$ and $\vec{n_0}$ (A). The sum of the connecting vectors $s\vec{p}_0$ and $s\vec{n}_0$ (B)+(C) is projected onto the supporting edge $\vec{e_s}$ (D). The projection is scaled by the conduction velocity in order to obtain the velocity of the snaxel (E).

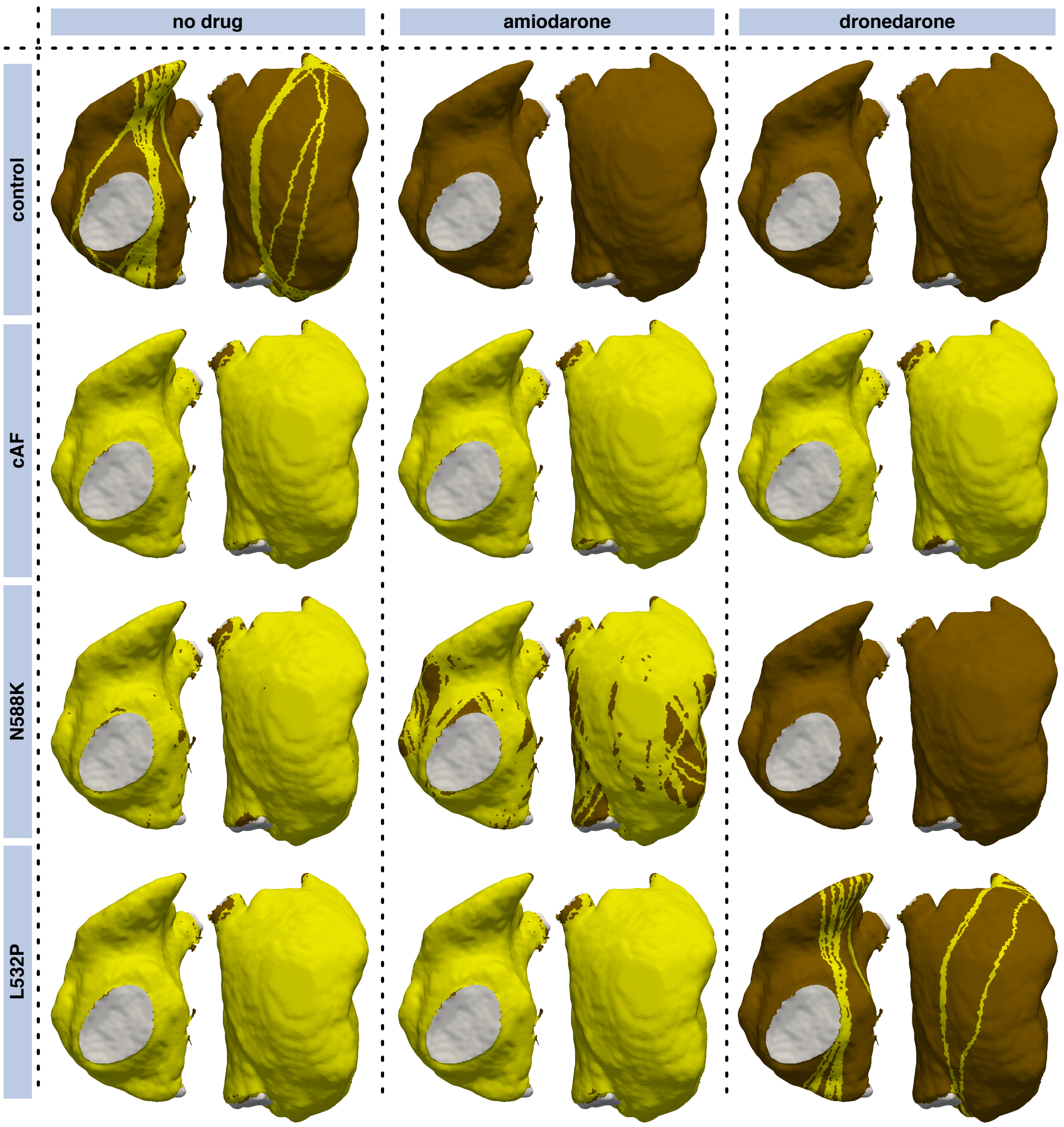

Figure 4: Vulnerability maps of the right atrium (RA) for combinations of different substrates and pharmacological agents. Besides a control substrate representing healthy myocytes, a chronic atrial fibrillation (cAF) remodeled substrate (neglecting changes of cell-to-cell coupling), and the two human ether-à-go-go-related gene (hERG) mutations N588K and L532P were evaluated. Standard concentrations of the antiarrhythmic agents amiodarone (2.3 M) and dronedarone (0.21 M) were administered in the center and right columns, respectively. Vulnerable pathways are marked in yellow on the RA myocardium (brown); the blood pool is indicated in gray. In contrast to Figure 10 in the paper, only the ERP was modeled substrate-specific, thus only considering differences in repolarization.

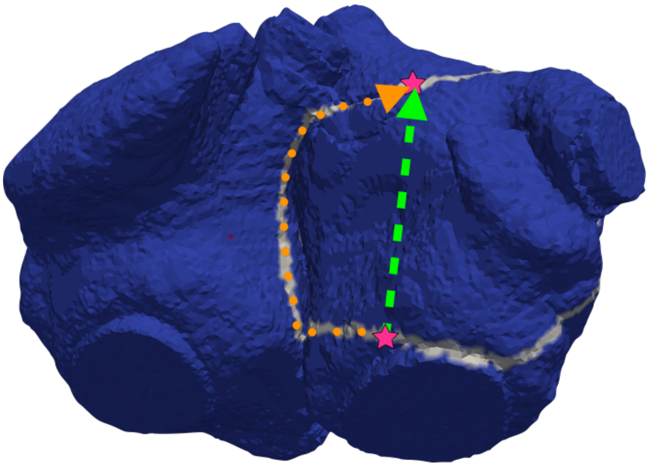

Figure 5: Limitation of geometrical snakes concerning biatrial loops. While a shortcut between the two purple stars exists (dashed green line), the snake cannot constrict further and remains on the dotted orange line due to the discrete interatrial connections.

3 SUPPLEMENTARY TABLES

Table 1. Coefficients of exponential curves representing the restitution of CV and ERP according to Equation (3) in the main manuscript and the anisotropy k according to Equation (2) in the main manuscript for different anatomical structures in the atria and different homogeneous substrates with and without the influence of amiodarone and dronedarone. For the homogeneous substrates, anisotropy was not changed. The CV for the homgeneous control model was reduced compared to the RA/LA tissue in the heterogeneous setup to obtain a similar total activation time.Parameters were estimated based on the output of monodomain tissue strand simulations. The resulting restitution curves are shown in Figure 3 in the main manuscript. Abbreviations: right atrium (RA), left atrium (LA), crista terminalis (CT), pectinate muscles (PM), Bachmann's bundle (BB), inferior isthmus (II), pulmonary veins (PV), right atrial appendage (RAA), left atrial appendage (LAA), tricuspid valve ring (TVR), mitral valve ring (MVR).

	CV				ERP		
	A (mm/s)	B (mm/s)	C (ms)	k	A (ms)	B (ms)	C (ms)
**RA/LA**	600.6	3.38×10^6	30.3	3.75	318.4	312.9	165.0
**scar**	0	0	1.0	1.00	318.4	312.9	165.0
**CT**	600.6	3.18×10^5	45.1	6.56	331.5	1000.0	94.0
**PM**	466.9	2.63×10^6	30.3	10.25	318.4	312.9	165.0
**BB**	656.6	3.69×10^6	30.3	9.00	318.4	312.9	165.0
**inferior isthmus**	600.6	3.38×10^6	30.3	1.00	318.4	312.9	165.0
**PVs**	600.6	1.41×10^5	40.7	3.75	276.1	55.8	915.4
**RAA/LAA**	600.6	4.10×10^6	29.9	3.75	302.7	92.3	224.0
**TVR/MVR**	600.6	1.74×10^6	23.9	3.75	256.8	128.9	305.4
**control**	453.6	2.55×10^6	30.3		318.4	312.9	165.0
**control (amio)**	373.3	1.38×10^9	18.2		351.3	232.3	285.2
**control (drone)**	430.9	6.02×10^9	23.7		411.9	1000.0	148.4
**cAF**	436.0	0	1		173.6	79.7	276.2
**cAF (amio)**	357.0	0	0		182.3	153.5	132.9
**cAF (drone)**	412.9	12.03	36.9		211.9	104.1	332.1
**N588K**	452.3	3.79×10^5	30.4		245.7	30.9	575.3
**N588K (amio)**	372.9	3.37×10^6	24.0		280.4	126.8	226.9
**N588K (drone)**	430.1	3.42×10^6	28.8		361.9	908.4	112.2
**L532P**	449.8	1.53×10^5	18.1		161.8	37.6	337.2
**L532P (amio)**	371.3	3.31×10^5	21.9		189.3	42.2	251.5
**L532P (drone)**	430.1	1.18×10^5	38.4		326.4	172.7	335.5

4 SUPPLEMENTARY REFERENCES

REFERENCES

Bischoff, S. and Kobbelt, L. P. (2004). Parameterization-free active contour models with topology control. *The Visual Computer* 20, 217–228

Colli Franzone, P., Guerri, L., and Rovida, S. (1990). Wavefront propagation in an activation model of the anisotropic cardiac tissue: asymptotic analysis and numerical simulations. *J Math Biol* 28, 121–176

Jacquemet, V. (2010). An eikonal approach for the initiation of reentrant cardiac propagation in reaction-diffusion models. *IEEE Transactions on Biomedical Engineering* 57, 2090–2098

Jacquemet, V. (2012). An eikonal-diffusion solver and its application to the interpolation and the simulation of reentrant cardiac activations. *Computer Methods and Programs in Biomedicine* 108, 548–558

Tomlinson, K. A., Hunter, P. J., and Pullan, A. J. (2002). A finite element method for an eikonal equation model of myocardial excitation wavefront propagation. *SIAM Journal on Applied Mathematics* 63, 324–350
